# Italy and the Comprehensive Agreement on Investments: disappointment over the process

**DOI:** 10.1007/s10308-021-00630-6

**Published:** 2021-07-25

**Authors:** Francesca Ghiretti

**Affiliations:** Istituto Affari Internazionali (IAI), Rome, Italy

## Abstract

The formal conclusion of the Comprehensive Agreement on Investments (CAI) has drawn much criticism. Criticisms for member states did not always recognise the same critical points. The case of Italy presents an instance in which the issue rather than laying in the content of the agreement was identified in the process. Not only had Italy been marginalised in the process of negotiation that led to the conclusion of the CAI, but also exponents from the government claim that leading negotiators, amongst which France and Germany, ignored Italy’s doubts about the deal when these were raised. Beyond politics, the paper shows that the Italian business community displays a response to the agreement in line with that of the rest of the EU in its positive assessment of the outcome.

## Introduction

The Comprehensive Agreement on Investments’ (CAI) formal conclusion between the European Union and the People’s Republic of China came at the very end of December 2020, after seven years of negotiations. Usually, in Brussels, the end of December is a relatively quiet time, as most people go on holiday and the institutions work at a reduced regime. However, 2020 demonstrated to be an exceptional year as the pandemic of Cov-Sars-2 disrupted the usual business until the very end; and the conclusion of the CAI fed into this exceptionalism. Admittedly, very few had predicted the achievement of the deal by the end of the year, if not at all. After the online EU-China summit held in September, even European Commission’s President Ursula Von der Leyen cautioned that plenty remained to be done for the deal to be concluded (Tiezzi, 2020).^1^ This was sufficient to confirm that despite the German presidency of the Council’s apparent desire^2^ to finalize the agreement, the deal was going to remain in the pipelines (Hanke Vela, 2020). Yet, sudden concessions on the Chinese side created momentum and opened the way for the deal’s conclusion (Godement, 2021).^3^

The agreement’s formal finalisation was not met with few criticisms, notably in reaction to China’s behaviour in 2020. Criticisms spanned from the suppression of voices that could have launched an early alarm of the pandemic, to the delaying of an international investigation on the origins of the virus, from the introduction of the national security law in Hong Kong to the (re)emergence of information regarding the situation in Xinjiang, from the so-called “wolf warrior” diplomacy to increased military exercises in the South China Sea and near Taiwan. These are only a few of the elements that saw China’s assertiveness further growing throughout 2020. Finally, most believed that following the election of a more like-minded US president, the EU would have waited and consulted with the new Biden administration before concluding such a deal with China.

Despite the formality, the deal is far from being concluded. The European Commission might have formally concluded the agreement, but it will need the European Parliament and the Council’s approval to make it effective. Considering the comments that followed the CAI announcement, it is not difficult to foresee lengthy negotiations with the European Parliament; the deal will not go through without intense scrutiny and negotiations. However interesting the European Parliament’s endeavours might be, it is the happenings within the Council that offer the perfect springboard to look at the focus of this brief paper, namely, how Italy positioned itself and reacted to the CAI. Officially, when asked, some Council members raised doubts about the deal, but none of them attempted to block it.

## Italy

Interestingly, of all the contentious elements mentioned above and others raised regarding China and the striking of the agreement, Italy was most concerned with how the deal came to be and how it was finalised. The two, the process and the conclusion, have in common that Italy’s role was marginalised. These were the main elements lamented by Ivan Scalfarotto, at the time, Italy’s undersecretary to foreign affairs (Fubini, 2020).^4^

To better understand Italy’s disappointment, we must take a step back to 2016/2017, when it looked as if Italy could join France and Germany and occupy a leading position within the EU. This fortunate situation for Italy resulted from two elements: the outcome of the Brexit referendum and the governmental setting of Italy. The announcement of the UK’s exit from the EU meant that the three big powers that for better or for worse had been balancing and shaping EU’s foreign policy, France, Germany, and the UK, suddenly found themselves scarce of one actor. The new setting was not a problem in se, but it was an opportunity for a country, such as Italy, which had been attempting to occupy a more important role at the EU level. In those years, Italy was governed by the Democratic Party (PD), a very pro-European party, and up to the end of 2016, the then Prime Minister Matteo Renzi had been trying to carve out a more prominent position for Italy in Europe and, arguably, in the world. Confidence in the success of the attempt was the result not only of the reformative and progressive spirit of the government with a relatively young and ambitious leader but also of the impressive victory PD led by Renzi brought home at the European elections of 2014 (40.8% of votes) (La Repubblica, 2014).^5^ For example, at the EU level, Renzi was the leading voice against guaranteeing China the market economy status (Robert, 2016).^6^ However, in 2017, under Renzi’s successor, Paolo Gentiloni, this potential new asset (France, Germany, and Italy) found a preliminary expression in the letter sent to the then European Commissioner Cecilia Malmström concerning the necessity to protect European assets from takeovers (European Parliament, 2019).^7^ Interestingly, in 2017, the undersecretary to that Ministry of Economic Development was precisely that same Ivan Scalfarotto who will follow the conclusion of CAI and who will lament Italy’s side-lining during the process that led to the finalisation of the agreement.

One might wonder what happened to the momentum created in 2016/2017 and Italy’s chances of joining the leading team. The impetus was short-lived as in 2018; elections brought a change of government and a shift in Italy’s approach towards the EU and China. The new government was a coalition formed by two populist parties, the Five Star Movement and League. Although this will change afterward, at the time, both shared an anti-European approach and the desire to diversify Italy’s partnerships beyond the EU. This soon translated into two connected actions that materialised in March 2020. First, the abstention of Italy at the vote for the adoption of the EU framework of investments^8^. Second, the state visit by Xi Jinping to Italy for the signing of a Memorandum of Understanding between the two countries that notably will make Italy the first G7 country to officially become part of the Belt and Road Initiative (BRI) (European Parliament, 2019). This latter decision will draw criticisms from Brussels and Washington.

In what could be seen as a clear reminder of who was steering the wheel, after the visit to Italy, President Xi went to France, where a similar format to that found in December 2020 awaited him; the then president of the European Commission, Jean-Claude Junker, the German Chancellor, Angela Merkel and the French President Emmanuel Macron.

In an interview to the Italian newspaper *Corriere della Sera* following the conclusion of CAI, Ivan Scalfarotto references the signing of the MoU with China as the mistake that led to Italy’s marginalisation by France and Germany in the negotiations for the CAI—and, one could add, more broadly in the EU. The former undersecretary highlights that while one of the intentions of signing the MoU was to guarantee a driving position to Italy in the relationship with China and thus, be viewed in Brussels as an asset in its negotiations with China, Italy obtained the opposite. It is perhaps more correct to view the result of the 2018 elections and the arrival of a government formed by populist and anti-European forces as the underlying reason for Italy’s marginalisation in EU affairs.

According to Scalfarotto, despite not being involved in the process of negotiations, in December 2020, Italy did not quietly accept the proposed format for the deal’s conclusion. It directly raised the issue that for the call with Xi Jinping, there should only be institutional representatives, namely, Von der Leyen for the Commission, Michel for the Council, and Merkel as Germany held the Presidency of the Council, but not French President Emmanuel Macron (Fubini, 2020).^9^ Thus, France and Germany showed Italy that it is a marginal actor as its remarks, if they were indeed raised, had been ignored. Italy was expectedly not alone in raising concerns regarding the process; many lamented to have been asked to give an opinion on CAI without having access to the entire draft of the deal (Šimalčík, 2021; Hanke Vela et al., 2021).^10^

In honour of the truth, Undersecretary Scalfarotto also criticised elements regarding the content of the deal. The former undersecretary mentioned the lack of protection for European investments, the non-acceptance of international courts for dispute settlement, the wrong signal sent concerning upholding EU’s values, and the mistake of not waiting for Washington as essential elements of concern. Nonetheless, there is little doubt that Italy’s lack of involvement in the process is the primary concern raised by Italy in the institutional voice of Ivan Scalfarotto. He, however, has also praised the conclusion of the deal as a significant achievement for European enterprises (Fubini, 2020).^11^

## Politics and business

As important as they can be, institutional voices are not the only voices that could be heard in the days of the announcement of the deal. Despite Italy’s usual situation where foreign policy and technical issues such as CAI are of no interest for the larger public and thus, receive only partial coverage, the matter did not pass by quietly.

The most critical voices raised were amongst politicians who usually are critical of China, mostly for China’s violation of human rights. One notable voice is undoubtedly that of Democratic Party’s MP Roberto Rampi, who, together with Forza Italia Senator Lucio Malan, is also a member of the Inter-Parliamentary Alliance on China. Rampi expressed his disapproval regarding the agreement’s conclusion, mostly about China’s human rights violations, highlighting how human rights is a matter that cannot be side-lined just because the EU is making an economic agreement (IPAC, 2021).^12^ On the same lines, but even more vocal is Ambassador Giulio Terzi, who has become a fierce voice against China in Italy and repeatedly criticized the CAI conclusion. In his case, the strongest reference is to human rights violations and the necessity for the EU not to “reward” a country that breaches fundamental human rights with an economic agreement (Terzi, 2021).^13^ However, Terzi, a former Ambassador to Washington, also highlighted the EU’s mistake in not coordinating with the USA.

Nonetheless, it is difficult to draw a clear map of where parties stand concerning the matter; single voices have been raised against and in favour of the deal. However, these were not from parties representative, and they were not delineating the official party line. This is not unusual in the latest Italian political scenario, the question of how to approach China has created divisions between and inside parties. Admittedly, however, in the case of CAI, the sparse emergence of voices results from a lack of national debate on the matter. Only a handful of politicians either directly involved or interested in China more broadly and businesses with an interest in the Chinese market, which is far from being the majority of Italian businesses followed the CAI.

It is precisely the Italian business sector that expressed a more positive assessment of the CAI. In line with most of the business’s voices around Europe, Italian companies welcomed China’s agreement as an opportunity. In the newspaper *Milano Finanza* (Milan Finance), Marco Marazzi, a partner at Baker and McKenzie, expressed his positive account of new possibilities for European and Italian businesses about the conclusion of the deal. According to Marazzi, Italy could obtain particular benefits from the CAI’s openings in the health sector (Marazzi, 2020).^14^ Later Marazzi, together with Mario Boselli, president of the Italy China Foundation, has written a piece on Milano Finanza titled “The Investment Agreement between the EU and China is a great opportunity for Italy”. In the article, the authors make a pledge to the new government for closer links with the country that alone is responsible for 30% of the global growth and that in 2025 will become the most important consumption market: China (Boselli & Marazzi, 2021).^15^ There, they mention other sectors in which Italian enterprises in China are strong and can grow, such as automotive, mechanics, fashion, chemicals, and alimentary. The two, however, lament the low levels of investments by Italian enterprises in China, which are around 10 billion per year, noting the difference with investments from Germany, 80 billion (Boselli & Marazzi, 2021).^16^ However, even positive assessments of the conclusion of the CAI, such as those mentioned above, recognise that not only Italian enterprises lag behind in investments in China compared to other European counterparts, but also that it will be difficult for CAI to change such a situation if before the change does not occur in the way investments are viewed in Italy. Investments are not detached from trade, and according to Marazzi and Boselli, currently, they remain the best method to gain market access and shares in China (Boselli & Marazzi, 2021).^17^

This short analysis proposed by Marazzi and Boselli touches upon a fundamental yet often overlooked issue with the CAI. The EU can make a superb deal, but it is up to member states and businesses to eventually grasp these opportunities. In Italy, this is far from being an assured outcome. In a country where 92%^18^ of the enterprises are small or medium enterprises, unless something changes in the way of thinking of both Italian businesses and the economic approach of the country by learning how to create networked systems, the advantages Italian enterprises could obtain from CAI risk to be very limited (Il Sole 24 Ore, 2019). The competitive Chinese market remains a challenging arena for Italian small and medium enterprises, regardless of CAI. However, indirectly, Italian enterprises could still benefit from a wider and easier access of EU’s actors into the Chinese market. It is no secret that Germany would be one of the main beneficiaries of CAI, and putting aside nationalistic discourses, Germany is Italy’s first trade partner (Il Sole 24 Ore, 2019).^19^ Hence, benefits to German economy and enterprises have the potential to bring benefits to the Italian economy too. That is particularly true for some key sectors of Italian export to Germany such as steel (and steel products), chemicals, industrial machines, transport, and electronics (infoMercatiEsteri, 2021).^20^

Italy's disappointment about how the negotiation and conclusion of the deal were held might have been stronger than that of other EU countries. Its recent history had fed the country with tangible hopes of a more proactive role on the European scene. However, internal political endeavours had made those hopes disappear. Furthermore, the attempt to gain a more prominent role by striking unusual partnerships did not pay off, not even when that same unusual partner, China, was on the other side of Brussels' negotiation table. The CAI conclusion showed to Italy, once more, that for the better, or the worse, when it comes to EU’s foreign policy, its voice is not more relevant than that of any other member state, exception made for France and Germany.

Nonetheless, if we leave the process aside and we look at content, the positive assessment made both by Ivan Scalfarotto and exponents of the Italian business word might have to be reconsidered or sized down. In reality, the grand majority of small and medium Italian enterprises is unlikely to benefit from CAI. Therefore, in theory, CAI could be an excellent opportunity for Italian and European enterprises. However, in reality, the number of enterprises that will benefit from this agreement might be significantly lower and focused on larger economic actors, which often do not include Italian companies.

## Footnotes

1 https://thediplomat.com/2020/09/china-eu-leaders-hold-intense-virtual-meeting/

2 https://www.politico.eu/article/europe-china-germany-weak-spot/

3 https://www.institutmontaigne.org/en/publications/wins-and-losses-eu-china-investment-agreement-cai

4 https://www.corriere.it/economia/finanza/20_dicembre_31/accordo-ue-cina-scalfarotto-italia-ignorata-inopportuna-presenza-macron-a4096114-4b36-11eb-9611-5c537b64e8e9_amp.html?__twitter_impression=true

5 https://www.repubblica.it/static/speciale/2014/elezioni/europee/italia.html

6 https://www.euractiv.com/section/trade-society/news/european-parliament-unites-against-chinese-mes/

7 https://www.europarl.europa.eu/RegData/etudes/BRIE/2018/614667/EPRS_BRI(2018)614667_EN.pdf

8 https://www.europarl.europa.eu/RegData/etudes/BRIE/2018/614667/EPRS_BRI(2018)614667_EN.pdf

9 https://www.corriere.it/economia/finanza/20_dicembre_31/accordo-ue-cina-scalfarotto-italia-ignorata-inopportuna-presenza-macron-a4096114-4b36-11eb-9611-

10 https://www.euractiv.com/section/eu-china/opinion/china-investment-agreement-and-the-golden-calf-of-eu-unity/; https://www.politico.eu/article/germanys-drive-for-eu-china-deal-draws-criticism-from-other-eu-countries/

11 https://www.corriere.it/economia/finanza/20_dicembre_31/accordo-ue-cina-scalfarotto-italia-ignorata-inopportuna-presenza-macron-a4096114-4b36-11eb-9611-5c537b64e8e9.shtml

12 https://twitter.com/ipacglobal/status/1349319047062675462

13 https://twitter.com/GiulioTerzi/status/1359269694805577728

14 https://www.milanofinanza.it/news/verso-un-accordo-ue-cina-sugli-investimenti-202012292222544020

15 https://www.milanofinanza.it/news/l-accordo-sugli-investimenti-ue-cina-e-una-grande-opportunita-per-l-italia-202102182219129066

16 https://www.milanofinanza.it/news/l-accordo-sugli-investimenti-ue-cina-e-una-grande-opportunita-per-l-italia-202102182219129066

17 https://www.milanofinanza.it/news/l-accordo-sugli-investimenti-ue-cina-e-una-grande-opportunita-per-l-italia-202102182219129066

18 https://www.infodata.ilsole24ore.com/2019/07/10/40229/

19 https://www.ilsole24ore.com/art/germania-primo-partner-commerciale-dell-italia-interscambio-2019-oltre-127-miliardi-ADuixyp; https://www.infomercatiesteri.it/scambi_commerciali.php?id_paesi=69

20 https://www.infomercatiesteri.it/scambi_commerciali.php?id_paesi=69#
